# Predictors of long-term success after successful explantation of continuous flow left ventricular assist device support

**DOI:** 10.1093/icvts/ivae091

**Published:** 2024-06-05

**Authors:** Takayuki Gyoten, Eisuke Amiya, Akihito Saito, Minoru Ono

**Affiliations:** Department of Cardiac Surgery, The University of Tokyo, Tokyo, Japan; Department of Cardiovascular Surgery, Saitama Medical University International Medical Center, Saitama, Japan; Department of Cardiovascular Medicine, The University of Tokyo, Tokyo, Japan; Department of Therapeutic Strategy for Heart Failure, The University of Tokyo, Tokyo, Japan; Department of Cardiovascular Medicine, The University of Tokyo, Tokyo, Japan; Department of Cardiac Surgery, The University of Tokyo, Tokyo, Japan

**Keywords:** Ventricular assist device, Mechanical circulatory support, Cardiac recovery, Dilated cardiomyopathy

## Abstract

**OBJECTIVES:**

Predictors and evaluations of continuous flow left ventricular assist device (cf-LVAD) explantation in recovered patients remain under discussion due to lack of evidence on long-term safety and efficacy. This study summarized our experiences regarding cf-LVAD explantation in non-ischaemic dilated cardiomyopathy patients and estimated a predictor for sufficient myocardial recovery allowing left ventricular assist device explant.

**METHODS:**

We retrospectively identified 135 adult patients with cf-LVAD therapy as bridge to heart transplant due to non-ischaemic dilated cardiomyopathy. Of those, 13 patients underwent device explantation (recovery group) after myocardial recovery. Twelve (92%) of the explanted patients were evaluated using our weaning protocol and underwent surgical explantation. Meanwhile, the remaining 122 continued with cf-LVAD therapy (non-recovery group).

**RESULTS:**

Multivariate logistic regression analysis revealed time interval between the first heart failure event and cf-LVAD implantation as an independent predictor for successful explantation. The optimal time interval cutoff value to predict cf-LVAD explantation was 7 months, with a sensitivity of 91.0% and specificity of 84.6%. Echocardiography in patients with successful cf-LVAD explantation showed significant improvement of left ventricular function and dimensions at 6 months postoperatively. The 13 explanted patients are currently alive at a median of 30 (interquartile range; 18–58) months after explantation. The survival rate free from rehospitalization due to heart failure following explantation was 100%. Left ventricular function and remodelling after explantation were also preserved.

**CONCLUSIONS:**

In non-ischaemic dilated cardiomyopathy patients with a short interval between the first heart failure event and cf-LVAD therapy, left ventricular myocardium may recover in an early phase after device implantation.

## Graphical Abstract

**Figure:**
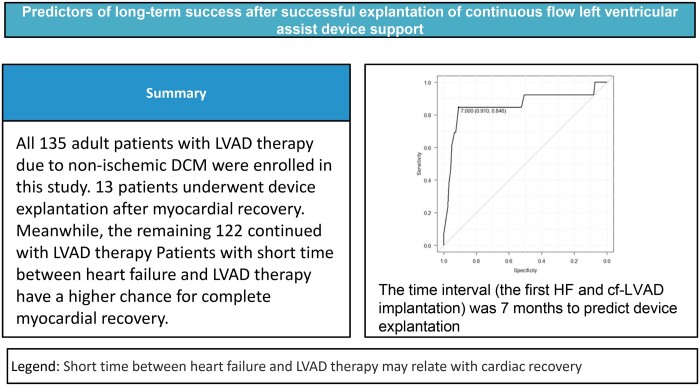

## INTRODUCTION

Continuous flow left ventricular assist device (cf-LVAD) therapy successfully improves survival, symptoms, exercise tolerance and quality of life in patients with advanced heart failure (HF) and long-term mechanical circulatory support (MCS) using left ventricular assist device (LVAD) has increasingly become a treatment option, especially as life-saving procedure for drug-refractory end-stage HF with severe LV dysfunction [1, 2]. The latest Interagency Registry for Mechanically Assisted Circulatory Support (INTERMACS) report shows an overall survival rate of 84% at 1 year after isolated LVAD implantation [3]. The EUROMACS data show a lower 1-year survival rate of only 70% [4]. LVAD support reduces rehospitalization due to HF, but in some cases may lead to ventricular assist device (VAD)-related complications, including infection, stroke, intestinal bleeding, right HF and VAD thrombosis, which may require intensive care treatment and invasive surgical therapy [2, 5, 6]. Treatment strategies using LVAD support are complex and associated with adverse effects that impose a significant burden on patients and caregivers and negatively affect resource utilization and overall public health [3–6].

cf-LVAD is used either as bridge to transplantation or destination therapy; it is well-known that some patients (1–2%) reach a bridge to recovery phase allowing cf-LVAD explantation [7]. The pathophysiology and mechanisms of LV myocardial recovery secondary to cf-LVAD support are neither well understood nor thoroughly explored; however, it is reported that LV unloading can boost LV reverse remodelling and lead to improvement of LV function in bridge to recovery patients [8–13].

Although the management of cf-LVAD explantation is still a debatable topic, cf-LVAD explantation should be positively considered when the haemodynamic status allows to do so after cf-LVD implantation. LVAD explantation seems meaningful in recovered patients, because it allows for the elimination of VAD-associated adverse events. Previously published case series supported cf-LVAD explantation in patients with myocardial recovery and showed excellent survival and outcomes at short-term follow-up [14, 15]. However, cf-VAD explantation in recovered patients is still controversial since it remains unclear whether recovery of heart function will be maintained [16].

This study aimed to summarize our cf-LVAD experiences and analyse the predictors for successful device explantation in patients with non-ischaemic dilated cardiomyopathy (DCM) with cf-LVAD.

## MATERIALS AND METHODS

### Data collection and follow-up

This single-centre study was approved by the institutional Ethics Committee of the University of Tokyo [3031-(4)]. We included all the patients from our institutional database qualifying for cf-LVAD implantation. All patients entered in the study period were offered participation in the study. Clinical decisions were made during interdisciplinary heart team conferences consisting of cardiologists, cardiac surgeons, perfusionists, cardio-anesthesiologists and VAD coordinators. As a bridge to transplant, a total of 191 patients were implanted with durable LVADs at our centre between November 2007 and April 2020 (Supplementary Material 1). Patients younger than 18 years of age (*n* = 7) and those with destination therapy strategy (*n* = 2) were excluded. Finally, a total of 135 non-ischaemic DCM patients were retrospectively included; all of whom were diagnosed with end-stage HF based on haemodynamic examination. A diagnosis of non-ischaemic DCM was obtained by myocardial biopsy histology. The devices included DuraHeart (Terumo Heart, Ann Arbor, MI, USA), EVAHEART (Sun Medical Technology Research Corp, Nagano, Japan), Jarvik 2000 (Jarvik Heart, New York, NY), HVAD (Medtronic, Minneapolis, USA), HeartMate II (Abbott Medical, Abbott Park, USA) and HeartMate 3 (Abbott Medical, Abbott Park, USA) devices. Demographics and clinical data before LVAD implantation included invasive pressures examination by right heart catheterization (RHC). As our standard strategy, clinical status was classified by INTERMACS levels, and patients with a diagnosis of level 1 (cardiogenic shock requiring temporary MCS) underwent Nipro paracorporeal pulsatile LVAD (Nipro-LVAD; Nipro, Osaka, Japan) implantation or Impella (Abiomed, Danvers, MA, USA) as a bridge to long-term MCS. We also collected imaging, laboratory values and cardiopulmonary exercise testing and surgical data. Transthoracic echocardiography (TTE) was regularly performed at 1, 6 and 12 months after LVAD implantation, and subsequently every 6 months. The clinical follow-up was closed on 31 May 2021, when the last enrolled patient had completed 1 year of follow-up.

### *Standardized weaning protocol in our institution to evaluate* ventricular assist device *explant candidates*

All potential candidates for VAD explantation were examined according to our three-phased institutional weaning protocol: selection of weaning candidates at clinic visit (first phase), LVAD speed reduction and saline loading test (second phase) and intraoperative outflow graft occlusion test (third phase). Workflow and details of this protocol are depicted in Supplementary Material 2 [17].

### Left ventricular assist device *explantation surgery approach*

Our standardized surgical LVAD explantation strategy consists of complete device removal (a remaining short segment of the outflow graft is oversewn) and LV apical linear closure. Our surgical strategy is summarized in a previously published case series [17].

### Follow-up

In the follow-up period, daily quality of life and functional status were assessed thoroughly. Finally, TTE and RHC were repeated to ensure sustained myocardial recovery. After cf-LVAD explantation, clinical follow-up and TTE were performed at 1, 6 and 12 months and yearly thereafter. Anti-coagulation was used during the first 6 months after LVAD explantation, and only aspirin was continued for life in patients after LVAD removal. A ‘successful’ cf-LVAD explantation was defined as the freedom from rehospitalization for HF and cardiovascular death during the follow-up period. Re-hospitalization for HF was defined as new-onset or worsening signs and symptoms of HF that require urgent therapy and hospitalization. The first HF event was defined as exacerbation of HF requiring intensive treatment by hospitalization.

### Statistical analysis

Results are expressed as a mean ± standard deviation or as median plus 25th–75th percentile interquartile range for continuous variables, and frequency and percentage for categorical variables where appropriate. Univariable comparisons were performed with Student’s unpaired *t*-test for continuous normally distributed data. Mann–Whitney *U*-test was used for comparisons of non-parametric data and Fisher’s exact test for categorical variables. The data in paired 2 groups were analysed using the signed Wilcoxon test. The paired Freedman test was used for comparisons of non-parametric data among paired 3 groups. Predictors of success following cf-LVAD explantation was evaluated using logistic regression analysis, and the results were expressed as odds ratios with 95% confidence intervals. Candidate covariates were chosen based on previous medical knowledge. Covariates were included via stepwise regression analysis using a probability for stepwise entry of 0.05. Receiver operating characteristic curves were plotted, and the area under the curves calculated to assess the optimal cutoff values for factors that predicted cf-LVAD explanation during LVAD support. The sensitivity and specificity values also were calculated.

Data for survival and freedom from cardiac events were derived using a Kaplan–Meier method; comparisons were made by a log-rank test. A *P*-value of <0.05 was considered statistically significant, and all reported *P*-values are two-sided. All statistical analyses were performed using R software (The R Project for Statistical Computing; The R Foundation).

## RESULTS

### Enrolled patients—cardiac recovery versus non-recovery

After the exclusion of non-DCM patients (ischaemic cardiomyopathy, *n* = 17; dilated hypertrophic cardiomyopathy, *n* = 16; drug-induced cardiomyopathy, *n* = 5; arrhythmogenic right ventricular cardiomyopathy, *n* = 3; sarcoidosis, *n* = 3; Endocardial fibrosis, *n* = 2, adult congenital, *n* = 2; valvular cardiomyopathy, *n* = 1), a total of 135 non-ischaemic DCM patients (*n* = 133, implantation in our hospital, and *n* = 2, in other institutions) were finally enrolled in this study (Supplementary Material 1). Of those, 13 patients (recovery group) underwent cf-LVAD explantation following myocardial recovery after a median of 10 months [interquartile range (IQR); 4–15 months] of cf-LVAD support. Twelve patients completed our standardized study protocol to test LVAD system explantation and proceeded to elective cf-LVAD explantation (Supplementary Material 2). One patient had thrombosis-related pump dysfunction 2 months after cf-LVAD implantation. Fortunately, left ventricular ejection fraction (LVEF) improved to within normal range, and urgent cf-LVAD explantation was performed without the evaluation.

### Perioperative, mid-term outcomes in cf-LVAD explanted patients

Perioperative outcomes are summarized in Table 1. None of the patients required temporary MCS such as extracorporeal membrane oxygenation (ECMO) or intra-aortic balloon pump (IABP) after cf-LVAD explanation, except 1 patient, who used IABP for potential haemodynamic deterioration.

**Table 1:** Short- and long-term outcomes in the recovery group

Variables	*N* = 13
Perioperative outcomes	
Extubation <24 h	6 (46%)
Intensive care unit stay (days), median (IQR)	4 (3–4)
In-hospital stay (days), median (IQR)	30 (23–36)
Medication at discharge	
Beta-blocker	13 (100%)
Angiotensin-converting enzyme inhibitor/angiotensin receptor blocker	13 (100%)
Mineralocorticoid receptor antagonist	9 (69%)
At discharge	
Home	13 (100%)
Hospital	0 (0%)
Rehabilitation	0 (0%)
Long-term follow-up	
All-cause mortality	0 (0%)
Major adverse cardiovascular events	0 (0%)
Cardiac death	0 (0%)
Cerebrovascular accident	0 (0%)
Re-ventricular assist device implantation	0 (0%)
Heart transplantation	0 (0%)
Re-admission due to heart failure	0 (0%)
Infection (sepsis)	0 (0%)

IQR: interquartile range.

In the perioperative period, no complications, including cerebrovascular accident, HF, bleeding and infection, occurred and all 13 patients survived and were discharged home. The median stay in the intensive care unit was 4 days (IQR; 3–4 days) and median total hospital stay was 30 days (IQR; 23–36 days).

During a median follow-up of 30 months (IQR; 18–56 months, range; 10–106 months), there was no cardiac- or non-cardiac-related death (Table 1). None of the patients required rehospitalization for recurrent left or new onset right HF, or for invasive treatment such as MCS and heart transplantation. Anti-coagulation therapy was stopped 6 months after cf-LVAD explantation in the absence of a specific medical indication to continue it, and no anticoagulation-associated complication occurred during the follow-up in our study cohort (Table 1). Overall survival after cf-LVAD explantation was 100% during the study period (a median follow-up of 30 months). Patients remained in NYHA functional class I with ongoing medical management (i.e. β-blocker and angiotensin-converting enzyme inhibitor or angiotensin receptor blocker).

### *Baseline characteristics and outcomes at* continuous flow left ventricular assist device *implantation*

Demographic and clinical features are shown in Table 2. Baseline characteristics were similar between the recovery group and the non-recovery group, except that the recovery group was more likely to have a higher body mass index (BMI) and a shorter time between first HF event and cf-LVAD implantation. None of the recovery group had cardiac resynchronization therapy with defibrillator (CRTD) before cf-LVAD implantation (*P* < 0.005). Beta-blocker dose at cf-LVAD implantation was significantly lower in the recovery group than in the non-recovery group (*P* = 0.011).

**Table 2:** Baseline characteristics in enrolled patients with recovery and non-recovery; *n* (interquartile range) if not otherwise specified

Variable	All, *n* = 135	Recovery group, *n* = 13	Non-recovery group, *n* = 122	*P*-value
Age (years), median (IQR)	42 (35–52)	37 (34–40)	43 (35–52)	0.097
Male gender	104 (77%)	10 (77%)	94 (77%)	1
Body mass index (kg/m^2^), median (IQR)	20 (18–22)	22 (20–25)	20 (18–22)	0.056
Body surface area (m^2^), median (IQR)	1.67 (1.53–1.76)	1.75 (1.57–1.82)	1.67 (1.53–1.75)	0.25
Hypertension	12 (9%)	3 (23%)	9 (7%)	0.084
History of stroke	23 (17%)	1 (8%)	22 (18%)	0.47
Chronic obstructive lung disease	2 (1%)	0 (0%)	2 (2%)	1
Diabetes mellitus	23 (17%)	4 (31%)	19 (16%)	0.24
Hyperlipidemia	17 (13%)	5 (38%)	12 (10%)	0.012
Chronic atrial fibrillation	27 (20%)	0 (0%)	27 (22%)	0.07
Prior cardiac resynchronization therapy	64 (47%)	0 (0%)	64 (52%)	<0.005
Prior intra-aortic balloon pump	20 (15%)	0 (0%)	20 (16%)	0.22
Prior open cardiac surgery	39 (29%)	2 (15%)	37 (30%)	0.35
Time between first heart failure event and LVAD implantation (months), median (IQR)	73 (32–121)	4 (2.0–7.0)	77 (43–132)	<0.005
Medication				
Beta-blocker	131 (97%)	12 (92%)	119 (98%)	1
Dose (carvedilol) (mg), median (IQR)	12.5 (5–25)	6.25 (1.25–10)	12.5 (5–25)	0.0105
Angiotensin-converting enzyme inhibitor/angiotensin receptor blocker	108 (80%)	8 (62%)	100 (82%)	0.25
Mineralocorticoid receptor antagonist	75 (56%)	9 (69%)	66 (54%)	0.23
Laboratory, median (IQR)				
Haemoglobin (g/dl)	11.2 (10.1–12.6)	11.6 (10.8–12.2)	11.2 (10.0–12.7)	0.58
Haematocrit (%)	34.1 (30.8–37.4)	34.7 (32.1–37.0)	34.1 (30.7–37.4)	0.8
Creatinine (mg/dl)	0.88 (0.72–1.12)	0.76 (0.65–1.02)	0.90 (0.72–1.13)	0.17
Creatinine clearance (ml/min)	72 (54–93)	87 (70–93)	69 (54–93)	0.18
Brain natriuretic peptide (pg/ml)	510 (218–784)	203 (124–626)	514 (235–795)	0.079
Aspartate aminotransferase (IU/l)	24 (19–33)	18 (15–24)	26 (20–34)	0.0083
Alanine aminotransferase (IU/l)	22 (15–35)	17 (11–27)	22 (15–36)	0.21
Total protein (g/dl)	6.6 (6.1–7.0)	6.2 (5.8–7.0)	6.6 (6.1–7.0)	0.29
Albumin (g/dl)	3.6 (3.2–4.0)	3.6 (3.2–3.8)	3.7 (3.2–4.0)	0.69
Lactate dehydrogenase (IU/l)	253 (209–317)	210 (169–290)	257 (211–317)	0.11
Total cholesterol (mg/dl)	151 (127–180)	180 (147–210)	150 (127–177)	0.044
Total bilirubin (mg/dl)	0.9 (0.7–1.5)	0.70 (0.50–1.1)	1.0 (0.70–1.5)	0.05
Direct bilirubin (mg/dl)	0.4 (0.2–0.6)	0.35 (0.18–0.43)	0.40 (0.20–0.60)	0.28
High-density lipoprotein (mg/dl)	47 (36–58)	43 (38–52)	47 (36–60)	0.68
Low-density lipoprotein (mg/dl)	85 (69–113)	100 (75–132)	84 (69–112)	0.17
Haemoglobin A1c (%)	5.7 (5.2–6.1)	5.9 (5.7–6.0)	5.6 (5.2–6.1)	0.41
Fasting blood sugar (mg/dl)	95 (84–111)	95 (91–118)	95 (84–111)	0.55
Electrocardiogram				
QRS (s)	0.12 (0.11–0.15)	0.11 (0.098–0.12)	0.13 (0.11–0.16)	0.043
QRS <0.12 s	59 (44%)	9 (69%)	50 (41%)	0.023
QTc (ms)	0.48 (0.45–0.52)	0.49 (0.48–0.51)	0.48 (0.45–0.52)	0.3
Respiratory function, median (IQR)				
%VC (%)	84 (70–95)	84 (78–87)	84 (70–95)	0.78
FEV1 (%)	80 (75–85)	88 (82–89)	79 (75–85)	0.11
DLCO (ml/min/mmHg)	68 (28–83)	82 (44–84)	68 (27–83)	0.58
INTERMACS level, median (IQR)	3 (2–3)	2 (2–3)	3 (2–3)	0.139
Level 1	2 (1%)	0 (0%)	2 (2%)	
Level 2	64 (47%)	9 (69%)	55 (45%)	
Level 3	66 (49%)	4 (31%)	62 (51%)	
Level 4	3 (2%)	0 (0%)	3 (2%)	
Temporary MCS				
Prior IABP	49 (36%)	7 (54%)	42 (34%)	0.225
Prior ECMO	5 (4%)	1 (8%)	4 (3%)	0.402
Prior Impella	3 (2%)	0 (0%)	3 (2%)	1
Prior extra-VAD implantation	23 (17%)	1 (8%)	22 (18%)	0.697
Redo-surgery	39 (29%)	2 (15%)	37 (30%)	0.346
Operative time (min), median (IQR)	395 (333–469)	338 (320–358)	407 (334–474)	0.021
Extracorporeal circulation time (min), median (IQR)	141 (101–167)	104 (97–116)	143 (101–173)	0.0195
Approach				1
Median	133 (99%)	13 (100%)	120 (98%)	
Lateral	2 (1%)	0 (0%)	2 (2%)	
Outflow position				1
Ascending aorta	133 (99%)	13 (100%)	120 (98%)	
Descending aorta	2 (1%)	0 (0%)	2 (2%)	
Implanted devices				0.586
DuraHeart	20 (15%)	2 (15%)	10 (8%)	
EVAHEART	40 (30%)	2(15%)	28 (23%)	
Jarvik 2000	36 (27%)	1 (8%)	22 (18%)	
HeartMate II	71 (53%)	6 (46%)	50 (41%)	
HeartMate 3	8 (6%)	2 (15%)	6 (5%)	
HVAD	9 (7%)	0 (0%)	6 (5%)	
Additional valve procedures				0.96
Aortic valve surgery	8 (6%)	2 (15%)	6 (5%)	
Mitral valve surgery	15 (11%)	2 (15%)	13 (11%)	
Tricuspid valve surgery	28 (21%)	5 (38%)	23 (19%)	
Aortic- and tricuspid valve surgery	2 (1%)	1 (8%)	1 (1%)	
Mitral- and tricuspid valve surgery	16 (12%)	0 (0%)	16 (13%)	
Aortic- and mitral- and tricuspid valve surgery	2 (1%)	0 (0%)	2 (2%)	

ECMO: extracorporeal membrane oxygenation; DLCO: diffusing capacity for carbon monoxide; FEV1: forced expiratory volume in 1 s; IABP: intra-aortic balloon pump; INTERMACS: Interagency Registry for Mechanically Assisted Circulatory Support; IQR: interquartile range; LVAD: left ventricular assist device; MCS: mechanical circulatory support; VAD: ventricular assist device; %VC: % vital capacity.

The median time from the first HF diagnosis to cf-LVAD implantation was 4 months (IQR; 2–7 months) in the recovery group and 77 months (IQR; 43–132 months) in the non-recovery group (*P* < 0.005). On ECG, QRS duration was shorter in the recovery group than in the non-recovery group (*P* = 0.043), and QRS <0.12 s was noted in 9 (69%) recovery patients. At the time of cf-LVAD implantation, distribution of INTERMACS status, the use of prior MCS, types of implanted cf-LVAD and concomitant surgical procedures were not significantly different between the 2 groups. TTE and RHC were performed to assess haemodynamic status before cf-LVAD implantation (Table 3). The parameters representing left ventricular dimension, including left ventricular end-diastolic diameter (LVDd), left ventricular end-systolic diameter (LVDs), left ventricular end-diastolic volume and LVEDS, were significantly larger in the non-recovery group than in the recovery group, resulting in the stroke volume not being statistically different. In addition, left atrial diameter was significantly larger in the non-recovery group. Other variables, such as LVEF and the grade of aortic regurgitation, mitral regurgitation and tricuspid regurgitation, were not significantly different between the 2 groups.

**Table 3:** Baseline results of transthoracic echocardiography and Swan-ganz catheterization; *n* (interquartile range) if not otherwise specified

Variable	All	Recovery group, *n* = 13	Non-recovery group, *n* = 122	*P*-value
Transthoracic echocardiography, median (IQR)				
LVEF (%)	15 (11–21)	21 (13–23)	15 (11–21)	0.19
FS (%)	7 (5–10)	10 (6.8–11)	7 (5.0–10)	0.099
LVDd (mm)	75 (65–81)	72 (61–73)	76 (66–82)	0.028
LVDs (mm)	69 (60–76)	64 (56–67)	70 (60–76)	0.021
IVS (mm)	7 (6–8)	7 (6–7)	7 (6–8)	0.3
LVPW (mm)	7 (6–8)	7 (7–8)	7 (6–8)	0.8
LVEDV (ml)	298 (218–362)	272 (185–281)	298 (224–364)	0.039
LVESV (ml)	243 (180–307)	213 (143–237)	255 (182–314)	0.019
LVSV (ml)	43 (32–63)	41(33–65)	43 (32–60)	0.89
LA (mm)	47 (41–55)	44 (39–46)	48 (41–56)	0.027
LVM (g)	224 (172–313)	200 (157–230)	238 (174–322)	0.096
AR, grade	0 (0–0)	0 (0–0)	0 (0–0)	0.51
MR, grade	2 (1–2)	1 (1–2)	2 (1–2)	0.36
TR, grade	1 (1–2)	1 (1–1)	1 (1–2)	0.42
RVSP (mmHg)	38 (28–51)	29 (21–42)	38 (28–52)	0.047
Swan-ganz catheterization, median (IQR)				
Mean right atrium (mmHg)	8 (5–12)	13 (5–16)	7 (5–11)	0.23
Systolic right ventricle (mmHg)	36 (27–46)	41 (28–46)	36 (27–46)	0.82
Diastolic right ventricle (mmHg)	9 (6–13)	9 (3–14)	9 (6–12)	0.9
Mean pulmonary capillary wedge pressure (mmHg)	20 (13–26)	25 (21–30)	20 (12–25)	0.16
Systolic pulmonary artery (mmHg)	38 (25–48)	42 (33–45)	38 (25–50)	0.9
Diastolic pulmonary artery (mmHg)	20 (14–26)	23 (18–31)	19 (14–25)	0.35
Mean pulmonary artery (mmHg)	27 (18–36)	32 (24–36)	27 (18–36)	0.54
Cardiac index (l/min/m^2^)	1.9 (1.56–2.24)	1.8 (1.16–2.45)	1.9 (1.57–2.21)	0.5
Cardiac output (l/min)	3.14 (2.65–3.76)	3.05 (2.03–4.03)	3.14 (2.70–3.74)	0.57
Pulmonary vascular resistance, wood	2.24 (1.42–3.33)	2.14 (1.10–2.91)	2.24 (1.44–3.53)	0.34
Right ventricular stroke work index (g m/beat/kg)	6.02 (3.82–8.21)	3.70 (3.18–5.49)	6.16 (3.93–8.21)	0.23
Pulmonary artery pulsatility index	2.32 (1.27–3.5)	1.11 (1.02–2.13)	2.48 (1.44–3.50)	0.16
Right atrial pressure/pulmonary capillary wedge pressure, ratio	0.43 (0.29–0.59)	0.50 (0.25–0.55)	0.41 (0.29–0.60)	0.88

AR: aortic valve regurgitation; FS: fractional shortening; IVS: interventricular septum; IQR: interquartile range; LVDd: left ventricular diastolic diameter; LVDs: left ventricular systolic diameter; LVEDV: left ventricular end-diastolic volume; LVEF: left ventricular ejection fraction; LVESV: left ventricular end-systolic volume; LVM: left ventricular mass; LVPW: left ventricular posterior wall; LVSV: left ventricular systolic volume; MR: mitral valve regurgitation; TR: tricuspid valve regurgitation.

### Haemodynamic response following LV unloading

**Figure 1:**
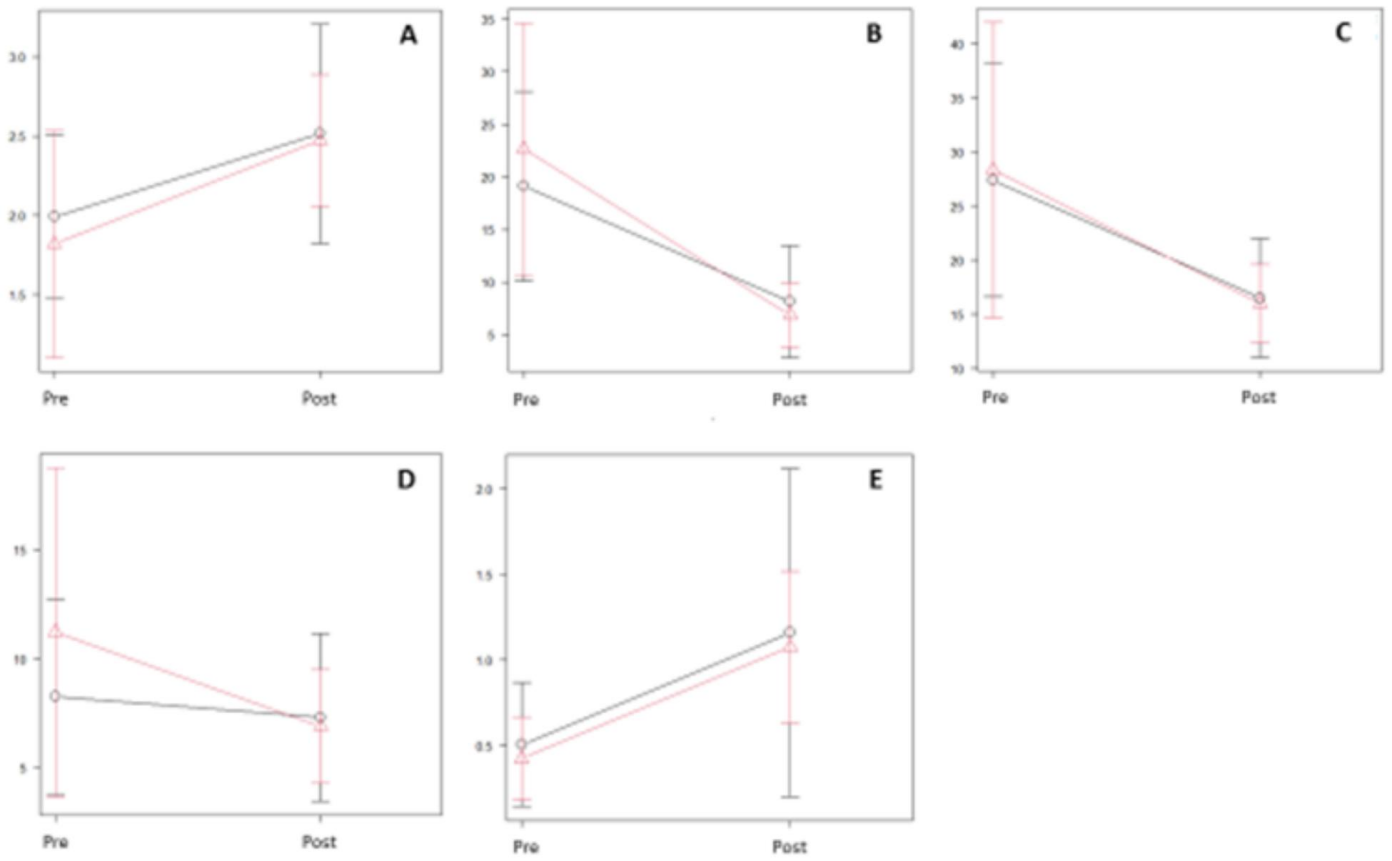
Comparison of haemodynamic parameters between pre- and 1-month post-left ventricular assist device implantation.

Haemodynamic parameters using RHC were compared between pre- and 1-month post-LVAD implantation. The differences in all parameters related to intracardiac pressure and cardiac output between the 2 groups before cf-LVAD implantation did not show statistical significance. Data are shown in Table 3. These values including pulmonary artery pressure, right atrial pressure, pulmonary capillary wedge pressure and cardiac index demonstrated a trend to improve 1-month post-LVAD implantation compared to pre-LVAD implantation (Fig. 1 and Table 4). This trend was observed in both the recovery group and the non-recovery group, and the values post-LVAD implantation were not significantly different between the 2 groups (Fig. 1 and Table 4).

**Table 4:** Comparison of haemodynamic parameters between pre- and 1-month post-left ventricular assist device implantation

	Recovery group, mean ± SD	Non-recovery group, mean ± SD	*P*-value
(A) Cardiac index (l/min/m^2^)			
Pre-LVAD implantation	1.82 ± 0.72	1.98 ± 0.52	0.42
Post-LVAD implantation at 1 month	2.55 ± 0.37	2.53 ± 0.65	0.93
(B) Mean pulmonary capillary wedge pressure (mmHg)			
Pre-LVAD implantation	23 ± 12	19 ± 9	0.20
Post-LVAD implantation at 1 month	7 ± 3	9 ± 5	0.23
(C) Mean pulmonary artery pressure (mmHg)			
Pre-LVAD implantation	29 ± 13	28 ± 11	0.69
Post-LVAD implantation at 1 month	16 ± 3	17 ± 6	0.57
(D) Mean right atrial pressure (mmHg)			
Pre-LVAD implantation	11 ± 7	8 ± 5	0.12
Post-LVAD implantation at 1 month	7 ± 3	7 ± 4	0.86
(E) Right atrial pressure/pulmonary capillary wedge pressure ratio			
Pre-LVAD implantation	0.42 ± 0.24	0.52 ± 0.41	0.48
Post-LVAD implantation at 1 month	1.11 ± 0.44	1.10 ± 0.93	0.95

(A) Cardiac index; (B) mean pulmonary capillary wedge pressure; (C) mean pulmonary artery pressure; (D) mean right atrial pressure; and (E) right atrial pressure/pulmonary capillary wedge pressure ratio (right atrial pressure/pulmonary capillary wedge pressure ratio).

LVAD: left ventricular assist device; post: post-LVAD implantation at 1 month; pre: pre-LVAD implantation; SD: standard deviation.

### Comparison of echocardiographic parameters in the recovery group versus the non-recovery group at long-term follow-up

**Figure 2:**
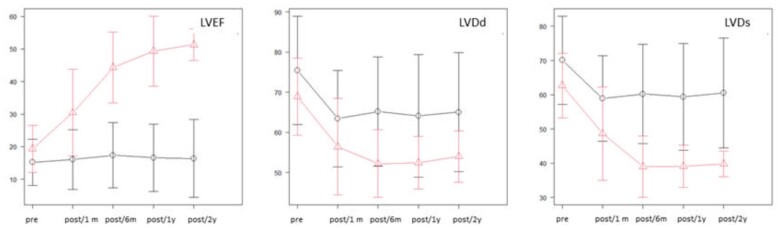
Comparison of echocardiographic parameters between the recovery and non-recovery groups.

Echocardiographic parameters (LVEF, LVDs and LVDd) of the 2 groups are depicted in Fig. 2 and Table 5. In the recovery group, the echocardiographic values after cf-LVAD explantation were also included in the calculation. LVEF improved within 1 month in the recovery group, compared to the non-recovery group, and the difference was statistically significant (27 ± 13 vs 16 ± 9, respectively, *p* < 0.005). Furthermore, the LVEF continued to improve over the following months, and the mean LVEF in the recovery group exceeded 40% at 6 months (Fig. 2 and Table 5). During the 2-year follow-up, this statistically significant difference was maintained at any given timepoint. The LV-diastolic and systolic diameters were significantly decreased after LV unloading using cf-LVAD in both 2 groups (Fig. 2 and Table 5). Moreover, the decrease in LVDd and LVDs was maintained in the recovery group compared to the non-recovery group, with a statistically significant difference at 6 months, 1 year and 2 years.

**Table 5:** Comparison of echocardiographic parameters between the recovery and non-recovery groups

	LVEF (%)	Recovery group, *n* = 13, mean ± SD	Non-recovery group, *n* = 122, mean ± SD	*P*-value	LVDd (mm)	Recovery group, *n* = 13, mean ± SD	Non-recovery group, *n* = 122, mean ± SD	*P*-value	LVDs (mm)	Recovery group, *n* = 13	Non-Recovery group, *n* = 122	*P*-value
Pre-LVAD support		18 ± 6	16 ± 7	0.32		67 ± 9	75 ± 13	0.044		61 ± 9	69 ± 13	0.030
Post-LVAD support at 1 month		27 ± 13	16 ± 9	<0.005		55 ± 11	63 ± 12	0.024		48 ± 12	59 ± 12	<0.005
Post-LVAD support at 6 months		43 ± 10	18 ± 10	<0.005		52 ± 10	64 ± 14	<0.005		39 ± 10	59 ± 15	<0.005
Post-LVAD support at 1 year		49 ± 10	16 ± 10	<0.005		53 ± 6	64 ± 14	0.016		39 ± 6	59 ± 15	<0.005
Post-LVAD support at 2 years		51 ± 5	16 ± 12	<0.005		54 ± 6	65 ± 15	0.030		40 ± 4	61 ± 16	<0.005

The number of patients performing LVAD explant was *n* = 0 at 1 month, *n* = 4 at 6 months, *n* = 8 at 1 year and *n* = 10 at 2 years after LVAD implantation, respectively.

LVAD: left ventricular assist device; LVDd: left ventricular diastolic diameter; LVDs: left ventricular systolic diameter; LVEF: left ventricular ejection fraction; post: post-LVAD explant; pre: pre-LVAD explant; SD: standard deviation.

### *Predictors for* left ventricular assist device *explantation*

Univariable and multivariable logistic regression analyses were performed to explore predictors of successful long-term LV reverse remodelling after cf-LVAD explantation. Pronounced associations were found between cf-LVAD explantation and BMI, ECG-QRS <0.12 ms, the value of B-type natriuretic peptide (BNP), LVDd at cf-LVAD implantation and the time interval between the first HF events and cf-LVAD implantation in the univariable logistic regression analysis (all *p* < 0.005, Table 5). Multivariable logistic regression analysis showed that the time interval between the first HF events and cf-LVAD implantation was an independent predictor for cf-LVAD explantation (odds ratio 0.97; 95% confidence interval 0.95–0.99, *p* = 0.0087, Table 6) The receiver operating characteristic curve analysis revealed that the optimal cutoff value of the time interval between the first HF events and cf-LVAD implantation to predict cf-LVAD explantation was 7 months, and the area under the curve was 0.859; this cutoff value had a sensitivity of 91.0% and specificity of 84.6% (Supplementary Material 3).

**Table 6:** Univariate and multivariate logistic regression analyses for the predictor of myocardial recovery after durable left ventricular assist device implantation

Variables	OR	95% CI	*P*-value	OR	95% CI	*P*-value
Age at LVAD implantation (years)	0.97	0.92–1.02	0.204			
Male gender	0.99	0.26–3.86	0.99			
Body mass index (kg/m^2^)	1.24	1.04–1.48	0.016			
QRS before LVAD implantation <0.12 s	6.12	1.27–29.60	0.024			
Brain natriuretic peptide (pg/ml)	0.998	0.99–1.00	0.0083			
Left ventricular diastolic diameter before LVAD implantation (mm)	0.95	0.90–0.99	0.043			
INTERMACS level	0.48	0.17–1.36	0.166			
Time between first HF and LVAD implantation (months)	0.97	0.95–0.99	0.0024	0.97	0.95–0.99	0.0075

CI: confidence interval; HF: heart failure; INTERMACS: Interagency Registry for Mechanically Assisted Circulatory Support; LVAD: left ventricular assist device; OR: odds ratio.

## DISCUSSION

In our study, we showed an extremely satisfying long-term survival rate (100% at median 3-year follow-up [IQR; 1–4 years]) and freedom from cardiac events (100% during the same period of follow-up) after cf-LVAD explantation in non-ischaemic DCM patients using our weaning protocol. Previous reports show that cardiac and physical functional capacity in patients with cf-LVAD explantation was well maintained compared to those with cf-LVAD therapy or heart transplantation [14, 17]. In the patients completing weaning protocol successfully, cf-LVAD explantation is feasible and an excellent long-term cardiac event free-survival seems to be achieved [18]. As increased mortality in on-going VAD patients is due to VAD-related complications, we believe that cf-LVAD explantation should be considered if possible.

Pan *et al.* [19] identified some predictors of myocardial recovery after LVAD implantation, which included younger age, female sex, lower BMI, non-ischaemic cause and short interval of HF before LVAD implantation. In our study, multivariate Cox regression analysis identified short interval between the first HF event and cf-LVAD implantation as the predictor with statistical significance (Table 6). In the present study, the median interval was 4 months (IQR; 2–7 months) in 13 patients with myocardial recovery. In our opinion, LV unloading with cf-LVAD support should be started as soon as possible in the event that HF could not be controlled with guideline direct medical therapy (GDMT) or CRTD implantation. However, currently, it may be difficult to implement this strategy since there is no supporting evidence and LVAD-related complications significantly increase morbidity and mortality.

In our study, 7 patients (54%) needed IABP and 1 patient (7.7%) ECMO before cf-LVAD implantation, who eventually underwent LVAD explantation. The severity of heart failure patients before LVAD implantation seems to be similar to that in other studies (14.15). Although these patients experienced acute haemodynamic deterioration, they may have had myocardial recovery during temporary MCS. This is because LV unloading with MCS is not only effective but allows for patients to be initiated on GDMT. Various temporary MCS strategies, such as IABP, ECMO and Impella, have been introduced. Of those, a percutaneous system with Impella 5.5 (Abiomed, Inc., Danvers, Massachusetts, USA) gained the attention of many physicians after its strong support of the circulatory system was observed [20]. Impella may be used as first choice for bridge to recovery since it can perform optimal LV unloading, reducing LV end-diastolic pressure and wall tension leading to a decrease in LV work and myocardial oxygen demand [20]. Therefore, we believe that successful myocardial recovery requires timeous and sufficient LV unloading and GDMT introduction in non-ischaemic DCM patients.

LVEF, LVEDD and LVESD significantly improved at 30 days compared with pre-LVAD (21 vs 27%, 72 vs 55 mm and 64 vs 46 mm, respectively; all median values, *P* < 0.05). This improvement in echocardiographic parameters reflecting myocardial recovery emerged in our patients within 30 days after LVAD implantation, compared to non-recovery patients, with the trend of improvement becoming more significant at 6 months (Fig. 2). Therefore, it would be reasonable to consider following up for further evaluation the patient in 6 months of the LVAD implantation as it would be useful to identify the explant candidate. In our study, 1 patient underwent LVAD explantation at 2 months following LVAD dysfunction due to pump thrombosis. Urgent explantation was successfully performed since the patient fortunately achieved some myocardial recovery within 1 month after LVAD implantation. The other 12 patients completed our weaning protocol in a stable condition.

Univariate Cox regression analysis showed that QRS duration, LVDd and BNP at LVAD implantation were significant predictors for successful explantation. None of the patients with successful cf-LVAD explantation had CRTD at implantation. In addition to echocardiographic parameters, ECG and the BNP value at LVAD implantation may also indicate a possibility of myocardial recovery. These markers may be associated with the severity of myocardial fibrosis [21]. Future prospective studies are needed to yield reliable markers for myocardial recovery during LVAD support.

There is no consensus on the optimal surgical strategy regarding LVAD explantation and management of postoperative antiplatelet and anticoagulant therapy. Certainly, surgical cf-LVAD removal itself may be associated with a risk of recurrent cardiac failure due to invasive redo open heart surgery. Therefore, as less invasive deactivation techniques, simplified surgical procedures ranging from driveline disruption to partial VAD explantation by thoracotomy with inflow occlusion using mechanical plugs have been reported [22]. In particular, this occlusion method mechanical using plugs seems to preserve cardiac geometry at the time of cf-LVAD removal and make easier the surgical approach [23–26]. However, the long-term safety and efficacy of these procedures are unclear since most studies have focused on short-term outcomes in a small study cohort. Additionally, anticoagulation or antiplatelet therapies may need to be continued since there is a risk of foreign material plug associated thrombosis [22]. Therefore, the life-threating risk of bleeding remains despite of the absence of LVAD support. In our study, among 13 patients performing cf-LVAD explantation, 7 (54%) patients had intracerebral bleeding (*n* = 3) or thrombosis (*n* = 4) during cf-LVAD support. However, no further bleeding or thrombotic complications occurred in patients after cf-LVAD explantation during the follow-up period.

Most importantly there is always a risk for recurrent HF, even if primary explantation was successful. Fortunately, myocardial recovery was preserved throughout the complete follow-up period (Fig. 2 and Table 5). All patients were treated with β-blocker and angiotensin-converting enzyme inhibitor/angiotensin receptor blocker during the study period. This GDMT is believed to be mandatory to preserve LV function after myocardial recovery [27]. Recently, sodium glucose cotransport-2 as heart failure therapy has been increasingly used with satisfying results [28]. Therefore, the effect of sodium glucose cotransport-2 use post-LVAD explantation should also be adequately studied.

### Limitations

This study has some limitations. This is a retrospective, single-centre observational study with a limited number of patients. Second, only non-ischaemic DCM patients were enrolled and analysed, but some DCM types such as DCM-like phenotypes may be included. Moreover, our TTE data were evaluated by experienced cardiologists, but not adjudicated by an external core lab. Thus, conclusions from our study should be taken with caution until confirmed by the currently ongoing multicentre clinical trial (ClinicalTrials.gov identifier NCT number: NCT03238690).

## Supplementary Material

ivae091_Supplementary_Data

## Data Availability

All data generated or analysed during this study are included in this published article and its supplementary information files.

## ABBREVIATIONS

BMI: Body mass index
BNP: B-type natriuretic peptide
cf-LVAD: Continuous flow left ventricular assist device
CRTD: Cardiac resynchronization therapy with defibrillator
DCM: Dilated cardiomyopathy
ECMO: Extracorporeal membrane oxygenation
GDMT: Guideline direct medical therapy
HF: Heart failure
IABP: Intra-aortic balloon pump
INTERMACS: Interagency Registry for Mechanically Assisted Circulatory Support
IQR: Interquartile range
LVAD: Left ventricular assist device
LVDd: Left ventricular end-diastolic diameter
LVDs: Left ventricular end-systolic diameter
LVEF: Left ventricular ejection fraction
MCS: Mechanical circulatory support
RHC: Right heart catheterization
TTE: Transthoracic echocardiography
VAD: Ventricular assist device
