# Supplementary material for: Predictors of long-term success after successful explantation of continuous flow left ventricular assist device support
Source: Interdiscip Cardiovasc Thorac Surg. 2024 Jun 5;38(6):ivae091. doi: 10.1093/icvts/ivae091 (PMC11878525; doi:10.1093/icvts/ivae091)
Supplement: ivae091_Supplementary_Data [file ivae091_supplementary_data.docx]

Suupl.1


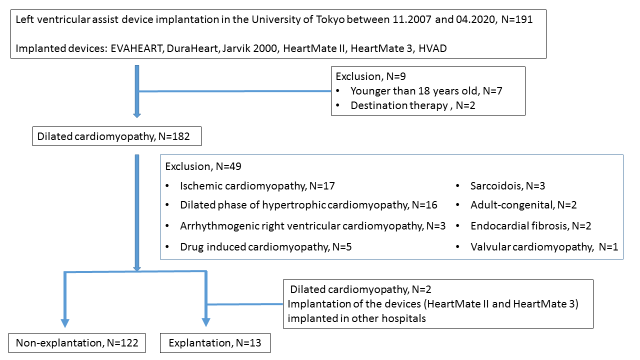


Suppl. 2


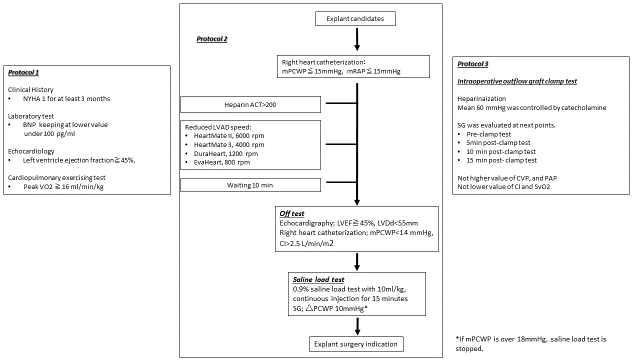


Suppl.3


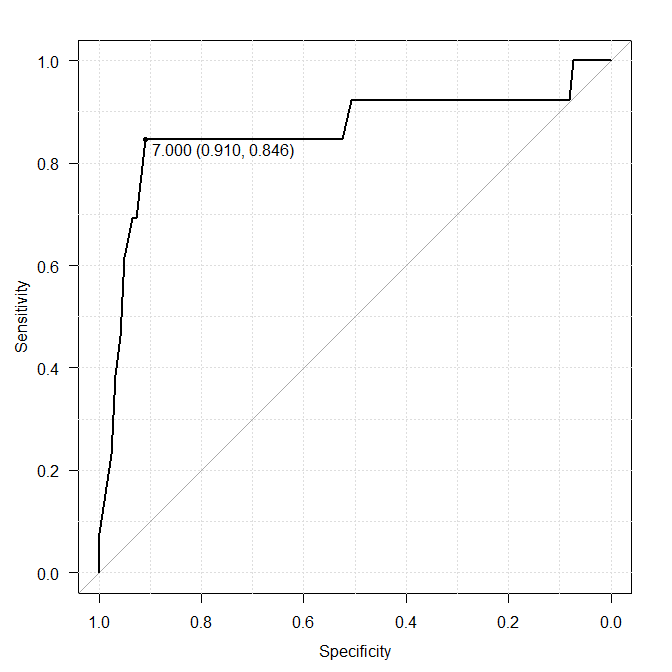


**Supplementary material:**

Suppl.1　Flow chart for selection of patients.

Suupl.2　Weaning protocol sequence.

NYHA = New York Heart Association, LVAD = left ventricular assist device, BNP = brain natriuretic peptide, mPCWP = mean pulmonary capillary wedge pressure, mRAP = mean right atrium pressure, CVP = central venous pressure, LVDd = left ventricular diastolic diameter, CI = cardiac index, PAP = pulmonary artery pressure, SvO2 = mixed venous oxygen saturation.

Suupl.3　A ROC curve plot showing an optimal cutoff value (7 months) of the time interval between the first HF events and cf-LVAD implantation for cf-LVAD explantation, with a sensitivity of 91.0% and specificity of 84.6%. The AUC is 0.859.
